# *Geobacter* Protein Nanowires

**DOI:** 10.3389/fmicb.2019.02078

**Published:** 2019-09-24

**Authors:** Derek R. Lovley, David J. F. Walker

**Affiliations:** Department of Microbiology, Institute for Applied Life Sciences, University of Massachusetts, Amherst, MA, United States

**Keywords:** pili, cytochrome, electron transfer, electromicrobiology, biomaterials

## Abstract

The study of electrically conductive protein nanowires in *Geobacter sulfurreducens* has led to new concepts for long-range extracellular electron transport, as well as for the development of sustainable conductive materials and electronic devices with novel functions. Until recently, electrically conductive pili (e-pili), assembled from the PilA pilin monomer, were the only known *Geobacter* protein nanowires. However, filaments comprised of the multi-heme *c*-type cytochrome, OmcS, are present in some preparations of *G. sulfurreducens* outer-surface proteins. The purpose of this review is to evaluate the available evidence on the *in vivo* expression of e-pili and OmcS filaments and their biological function. Abundant literature demonstrates that *G. sulfurreducens* expresses e-pili, which are required for long-range electron transport to Fe (III) oxides and through conductive biofilms. In contrast, there is no definitive evidence yet that wild-type *G. sulfurreducens* express long filaments of OmcS extending from the cells, and deleting the gene for OmcS actually *increases* biofilm conductivity. The literature does not support the concern that many previous studies on e-pili were mistakenly studying OmcS filaments. For example, heterologous expression of the aromatic-rich pilin monomer of *Geobacter metallireducens* in *G. sulfurreducens* increases the conductivity of individual nanowires more than 5,000-fold, whereas expression of an aromatic-poor pilin reduced conductivity more than 1,000-fold. This more than million-fold range in nanowire conductivity was achieved while maintaining the 3-nm diameter characteristic of e-pili. Purification methods that eliminate all traces of OmcS yield highly conductive e-pili, as does heterologous expression of the e-pilin monomer in microbes that do not produce OmcS or any other outer-surface cytochromes. Future studies of *G. sulfurreducens* expression of protein nanowires need to be cognizant of the importance of maintaining environmentally relevant growth conditions because artificial laboratory culture conditions can rapidly select against e-pili expression. Principles derived from the study of e-pili have enabled identification of non-cytochrome protein nanowires in diverse bacteria and archaea. A similar search for cytochrome appendages is warranted. Both e-pili and OmcS filaments offer design options for the synthesis of protein-based “green” electronics, which may be the primary driving force for the study of these structures in the near future.

## Introduction

The concept of long-range electron transport through microbially produced protein filaments with nanometer diameters (Reguera et al., 2005) has provided new insights into microbial physiology and ecology (Lovley, 2017b,c,d). Protein nanowires can extend the potential distance of electronic interaction between cells and their environment, facilitating electron exchange with insoluble minerals and other cells. Enhancing the expression of protein nanowires is a strategy for increasing biofilm conductivity and current production in bioelectrochemical devices (Lovley, 2017b). Protein nanowires produced by microbes or fabricated *in vitro* with microbe-inspired designs, are “green,” sustainable electronic materials with physical properties and potential for functionalization that expand potential applications beyond those feasible with other nanowire materials (Lovley, 2017a,b; Creasey et al., 2018; Gutermann and Gazit, 2018; Ing et al., 2018; Ueki et al., 2019). A wide diversity of microorganisms appear to produce protein nanowires (Reguera et al., 2005; Holmes et al., 2016; Sure et al., 2016; Tan et al., 2017; Walker et al., 2018a,b, 2019), but in most instances, their structure or function have not yet been intensively investigated.

The protein nanowires expressed by *Geobacter* species are of interest because of the environmental and practical significance of microbes in this genus. As previously reviewed in detail (Lovley, 2011, 2017d; Lovley et al., 2011; Mahadevan et al., 2011), *Geobacter* species play an important role in the global carbon cycle because they are often the most abundant Fe(III)-reducing microorganisms in soils and sediments in which Fe(III) reduction is an important terminal electron acceptor for organic matter oxidation. *Geobacter* species also are effective in the bioremediation of organic (Lovley et al., 1989; Anderson et al., 1998; Rooney-Varga et al., 1999) and metal (Lovley et al., 1991; Lovley, 1995, 2003; Anderson et al., 2003) contaminants in subsurface environments. Oxidation of organic compounds by *Geobacter* species coupled to direct interspecies electron transfer (DIET) to methanogens is an important aspect of carbon and electron flux in some methanogenic soils (Holmes et al., 2017) and anaerobic digesters converting organic wastes to methane (Morita et al., 2011; Rotaru et al., 2014). Stimulating *Geobacter*-enabled DIET with the addition of conductive materials is an effective strategy for accelerating and stabilizing anaerobic digestion (Lovley, 2017c, 2017d; Martins et al., 2018).

The most intensively studied *Geobacter* protein nanowires are electrically conductive pili (e-pili). As detailed in this review, a substantial number of studies have demonstrated that PilA, the type IV pilin of *G. sulfurreducens* (Reguera et al., 2005), assembles into e-pili (Table 1). Furthermore, diverse mutant strains, constructed to evaluate the *in vivo* function of e-pili, have consistently yielded phenotypes consistent with the need for PilA pilin-based e-pili for long-range electron transport (Table 2). More recently, it has been discovered that the *G. sulfurreducens* multi heme outer surface *c*-type cytochrome OmcS can assemble into filaments (Filman et al., 2019; Wang et al., 2019). One report, Wang et al. (Wang et al., 2019) made the remarkable claims that: (1) previous studies on *G. sulfurreducens* e-pili had made a mistake, and were in fact studying filaments of OmcS; (2) OmcS filaments, not e-pili, were the primary conduits for long-range electron transport in *G. sulfurreducens*; and (3) the OmcS filament structure “explains the remarkable capacity of soil bacteria to transport electrons to remote electron acceptors”.

**Table 1:** Evidence that the PilA pilin monomer of *Geobacter sulfurreducens* assembles into electrically conductive pili (e-pili).

Observation	References
Heterologous expression of e-pili from *G. sulfurreducens* PilA pilin monomer in *Pseudomonas aeruginosa*	(Liu et al., 2019)
e-Pili in preparations from *G. sulfurreducens* in which PilA was the only protein recovered and no iron or cytochrome spectra detected	(Cologgi et al., 2011; Lampa-Pastirk et al., 2016)
Scanning tunneling microscopy revealed conductive cell-associated pili without electronic states expected for cytochromes	(Veazey et al., 2011)
The only filaments emanating from a strain expressing PilA modified with peptide tags contain the peptide tags and are conductive	(Ueki et al., 2019)
e-Pili conductivity can be tuned up or down by heterologous expression of PilA monomer genes with different aromatic amino acid contents	(Vargas et al., 2013; Liu et al., 2014; Adhikari et al., 2016; Lampa-Pastirk et al., 2016; Steidl et al., 2016; Tan et al., 2016a,b, 2017)
Filaments with a diameter consistent with e-pili (3 nm) emanating from cells propagate charge, but filaments expressed in a strain expressing a PilA monomer with reduced aromatic amino acid content do not	(Malvankar et al., 2014)

**Table 2:** Evidence that e-pili, but not OmcS filaments, are involved long-range electron transport in *Geobacter sulfurreducens*.

Observation	References
Deleting gene for OmcS *increases* biofilm conductivity and has no impact on production of high current densities	(Nevin et al., 2009; Malvankar et al., 2012)
Strain KN400 which expresses very little OmcS, but abundant e-pili produces biofilms with higher conductivity and generates higher current densities	(Yi et al., 2009; Malvankar et al., 2011, 2012)
Expressing pili with lower conductivities decreases current density even though OmcS abundance is not affected	(Vargas et al., 2013; Liu et al., 2014; Steidl et al., 2016; Tan et al., 2016b)
Cells expressing abundant OmcS, but poorly conductive pili are ineffective in Fe(III) oxide reduction	(Vargas et al., 2013; Liu et al., 2014; Tan et al., 2016b)
Nano-magnetite additions rescue *omcS*-deletion mutant to restore capacity for Fe(III) oxide reduction, but cannot rescue *pilA* deletion mutant	(Liu et al., 2015)
*G. uraniireducens,* which possesses an OmcS homolog, but produces poorly conductive pili, reduces Fe(III) oxide with an electron shuttle and produces low current densities	(Rotaru et al., 2015; Tan et al., 2016b)

**Figure 1:**
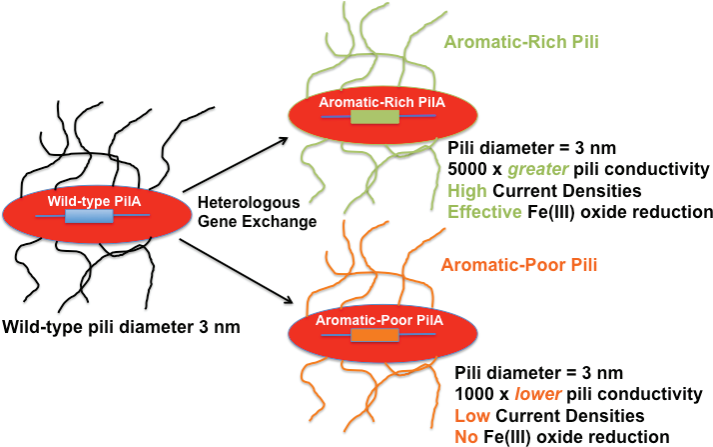
Heterologous expression of PilA pilin monomer genes in *G. sulfurreducens* yields pili with vastly different (10^6^-fold) conductivities of the individual wires. The pili in the strains with heterologously expressed pilin monomer genes have a diameter that is the same as wild-type pili (3 nm) and inconsistent with the diameter of OmcS filaments (4 nm). Reducing the conductivity of the pili has a dramatic negative impact on phenotypes for extracellular electron transport. The results summarized (Vargas et al., 2013; Tan et al., 2017) are just two examples of many on heterologous expression (Liu et al., 2014; Steidl et al., 2016; Tan et al., 2016b) that have reached similar conclusions.

It is difficult to reconcile these claims with the abundant evidence for e-pili existence and function in extracellular electron transfer (Tables 1, 2). As detailed in this review, multiple lines of evidence rule out the possibility that filaments identified as e-pili in many previous studies were actually OmcS filaments. A simple example is the heterologous expression of different PilA pilin monomer genes in *G. sulfurreducens* (Figure 1). The filaments expressed retained the diameter of e-pili, not OmcS, and differed greatly in conductivity, depending on the density of aromatic amino acids in the pilin monomer. The strains expressing poorly conductive pili were severely limited in long-range electron transport. How could changes in the aromatic density of the PilA monomer influence the conductivity of individual OmcS filaments?

The claim for OmcS as the primary conduit is inconsistent with the results of many previous studies, which considered the possibility of OmcS filaments (Table 2). Just one example among many is the finding that strains in which the OmcS gene was deleted had *higher* biofilm conductivities than the biofilm of the wild-type in which OmcS was abundant (Malvankar et al., 2012). The claim that the OmcS filament structure explains long-range electron transport in soil bacteria is inconsistent with the fact that OmcS is not widely found in the microbial world. Even many *Geobacter* species, including *Geobacter metallireducens*, the closest known relative of *G. sulfurreducens*, lack OmcS (Butler et al., 2010). *Geobacter uraniireducens*, which has an OmcS homolog (Butler et al., 2010) but lacks e-pili (Tan et al., 2016b), has phenotypes inconsistent with filament-based long-range electron transport (Butler et al., 2010; Rotaru et al., 2015; Tan et al., 2016b). It is incapable of producing high current densities on electrodes (Rotaru et al., 2015) and reduces Fe(III) oxide *via* production of a soluble electron shuttle (Tan et al., 2016b). OmcS is important for some forms of *G. sulfurreducens* extracellular electron exchange (Mehta et al., 2005; Summers et al., 2010; Tang et al., 2019). An important question is whether OmcS exists as filaments extending from the cells *in vivo* or whether OmcS attached to cells or e-pili is the functionally important localization of OmcS.

The purpose of this review is to assemble and evaluate the available evidence on the *in vivo* expression of e-pili and OmcS and their biological function in *G. sulfurreducens.* Hopefully, basic principles developed from the intensive studies on this one microbe will have transfer value to a greater diversity of other microbes that may also rely on protein nanowires for extracellular electron exchange. The focus is on biological function. Details of the mechanisms for electron transport along the filaments will not be discussed in depth because: (1) this topic is a matter of debate, requiring further experimentation (Lovley, 2017b; Creasey et al., 2018; Ing et al., 2018; Ru et al., 2019); and (2) it is not necessary to know the fine-scale mechanistic details of electron transport along the filaments in order to identify the filaments or evaluate their biological function. The primary questions addressed are: (1) what is the evidence for e-pili and OmcS filaments in live cells? and (2) what is the evidence for a physiologically relevant role of e-pili and/or OmcS filaments in long-range electron transport?

### Discovery of Electrically Conductive Pili

From the earliest studies (Gorby and Lovley, 1991; Lovley et al., 1993), to the present, investigations on the mechanisms for extracellular electron exchange in *Geobacter* species have focused primarily on cytochromes (see Lovley et al., 2004, 2011; Lovley, 2006; Shi et al., 2016, for a chronological review). Most of these studies focused on *c*-type cytochromes localized in the periplasm and outer membrane.

The impetus for expanding the investigation of extracellular electron transport mechanisms beyond cell-associated cytochromes was the finding that *G. metallireducens* expressed flagella and pili when grown on insoluble Fe(III) or Mn (IV) oxides, but not when grown with soluble Fe(III) citrate (Childers et al., 2002). Transcripts for the gene for the putative pilin monomer, PilA, were detected during growth on Fe(III) oxide, but not soluble Fe(III) (Childers et al., 2002). This same pattern of pili expression during growth on Fe(III) oxide, but not with soluble Fe(III), was also observed in *G. sulfurreducens* (Reguera et al., 2005). The diameter of the *G. sulfurreducens* filaments measured in these initial studies was 3 nm, consistent with later measurements of *G. sulfurreducens* e-pili diameter (Malvankar et al., 2014, 2015; Adhikari et al., 2016; Tan et al., 2017). As detailed below, heterologous expression of the *G. sulfurreducens* PilA pilin monomer in *Pseudomonas aeruginosa* also yields e-pili with a diameter of 3 nm (Liu et al., 2019). The diameter of OmcS filaments is 4 nm (Filman et al., 2019; Wang et al., 2019). The 3 nm diameter of the *G. sulfurreducens* pili is thinner than that of other bacteria, presumably because the *G. sulfurreducens* pilin monomer is comprised of only 61 amino acids, lacking the large “head group” that is typically displayed on the outer surface of other type IV pili (Reguera et al., 2005; Malvankar et al., 2015). Although it was possible to evolve strains of *G. sulfurreducens* to produce filaments other than PilA pilin-based pili (Klimes et al., 2010), this evolved strain was not included in any studies of e-pili. No filaments were observed when the gene for the PilA pilin monomer was deleted from the wild-type strain (Reguera et al., 2005). The simplest explanation for these results was that the filaments in the wild-type strain of *G. sulfurreducens* were pili comprised of PilA pilin monomer.

**Figure 2:**
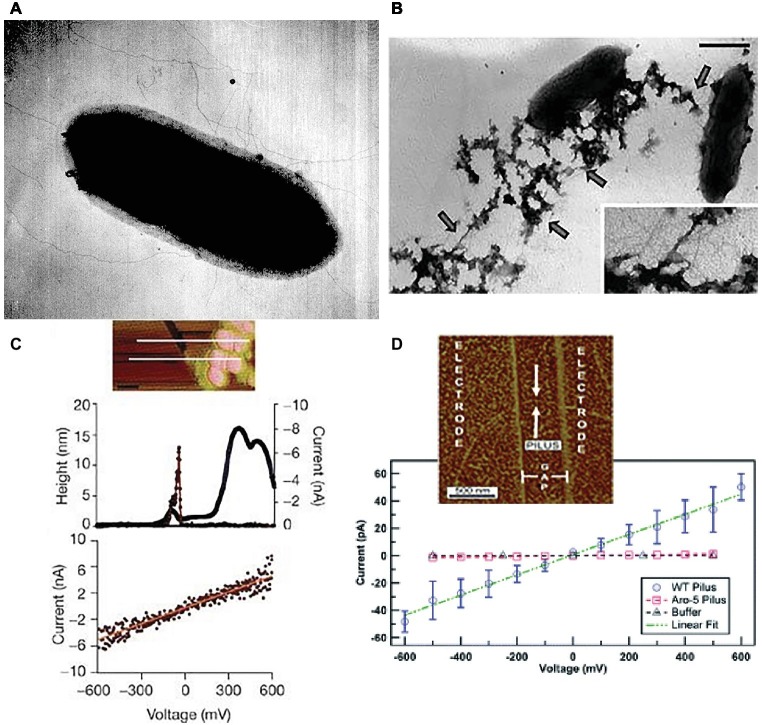
*Geobacter sulfurreducens* pili characterization. **(A)** Early (circa 2002) transmission electron micrograph of cell expressing pili. **(B)** Transmission electron micrograph of Fe(III) oxide attached to pili, scale bar = 0.5 μm. **(C)** Top, topographic atomic force microscope (AFM) image of pilus and unidentified additional material sheared from the outer cell surface deposited on highly oriented pyrolytic graphite (HOPG). Middle, topographic and current traces from conductive tip along the top cross-section shown above. Bottom, Current response to applied voltage from the conductive AFM tip. Current flows between the tip and the HOPG through the pilus. **(D)** Current-voltage response along the length of individual pili from wild-type and strain Aro-5 strains of *G. sulfurreducens* that were deposited on nano-electrode arrays. Image in **(A)** from laboratory archives. Images in **(B,C)** reproduced with permission from (Reguera et al., 2005). Images in **(D)** reproduced with permission from (Adhikari et al., 2016).

In addition to growth on Fe(III) or Mn(IV) oxides, pili expression could also be induced in *G. sulfurreducens* by growing it at the suboptimal temperature of 25°C with fumarate as the electron acceptor (Reguera et al., 2005). When poorly crystalline Fe(III) oxide was added to these fumarate-grown cells, the nanoparticles (ca. 30 nm) preferentially attached to the pili (Figure 2). This observation led to the question of how could the cell transfer electrons to Fe(III) oxides if they were suspended on pili at such a long distance from the cell surface. Could the pili be conductive? Conductive-tip atomic force microscopy (AFM) revealed (Figure 2) that the pili were electrically conductive with an ohmic-like (current = voltage/resistance) linear current-voltage response (Reguera et al., 2005). These findings, coupled with the result that the *pilA*-deficient mutant could not reduce Fe(III) oxides, lead to the conclusion that the pili of *G. sulfurreducens* function as a “microbial nanowire” for long-range extracellular electron transfer (Reguera et al., 2005). Analysis of current production densities on the anodes of bioelectrochemical devices, suggested that the microbial nanowires also played an important role in long-range electron transport through *G. sulfurreducens* biofilms producing high current densities (Reguera et al., 2006). Pilin-based microbial nanowires are now referred to as e-pili to avoid confusion with other conductive structures (Lovley, 2017b).

### Discovery of OmcS and OmcS Filaments

Like e-pili, the six-heme *c*-type cytochrome OmcS was discovered by comparing protein expression in cells grown on insoluble Mn(IV) or Fe(III) oxides versus soluble Fe(III) citrate (Mehta et al., 2005). OmcS was abundant in proteins easily sheared from the outer surface of cells grown on Mn(IV) oxide. OmcS gene transcripts were detected with RT-PCR during growth on Fe(III) oxide and fumarate, but not Fe(III) citrate (Mehta et al., 2005).

As with e-pili, deletion of the gene for OmcS prevented growth on Mn(IV) and Fe(III) oxides, but growth with soluble extracellular electron acceptors was not impacted (Mehta et al., 2005). The outer surface localization of OmcS, coupled with the previously observed localization of Fe(III) oxide on pili, led to the suggestion that OmcS was an important conduit between membrane-spanning *c*-type cytochromes that transported electrons to the outer surface and the e-pili (Mehta et al., 2005; Lovley, 2006). As detailed in a subsequent section, OmcS has been localized on the outer cell surface as well as filaments (Leang et al., 2010, 2013; Flanagan et al., 2017). The possibility that OmcS might form filaments to facilitate long-range electron transport at distance from the cell was considered in several studies, but eventually ruled out (Tran, 2009; Leang et al., 2010).

However, examination of the filaments in preparations of outer surface *G. sulfurreducens* proteins with cryo-electron microscopy revealed that OmcS can assemble into μm-long filaments (Filman et al., 2019; Wang et al., 2019). The fact that OmcS filaments were observed in concentrated protein assemblages, rather than emanating from the cells that were the source of the outer-surface proteins is important because one possibility is that the OmcS filaments could assemble as an artifact generated during the harvesting of the outer-surface proteins. During purification the highly hydrophobic OmcS (Qian et al., 2011) and other proteins are separated from the lipid-rich environment of the outer-cell surface and suspended in high salt and high pH aqueous solutions. Changes in chemical conditions are known to induce *c*-type cytochrome monomers to polymerize into nanowires (Hirota et al., 2010; Haldar et al., 2015; Alvarez De Eulate et al., 2017; Hirota, 2019; Nucara et al., 2019). An important stabilizing element in the OmcS filament structure is the coordination of a histidine in one subunit with the iron in the heme of an adjacent subunit (Filman et al., 2019; Wang et al., 2019). The his-heme metal coordination is a very strong bond that can form spontaneously, as observed in the routine purification of proteins modified with a His-tag (Bornhorst and Falke, 2000). The coordination between histidine and metals is favored at higher pH (Bornhorst and Falke, 2000), such as that used in the outer surface filament preparation methods that recovered OmcS filaments. As discussed in more detail in subsequent sections, OmcS filaments emanating from wild-type *G. sulfurreducens* have not yet been imaged, but some evidence (Wang et al., 2019) is indicative of OmcS filament expression by a mutant strain that greatly overexpressed OmcS.

### Documented Assembly of Pilin Monomers Into Electrically Conductive-Pili

The potential for pilin monomers to assemble into e-pili can clearly be demonstrated in microorganisms that do not express outer surface cytochromes. This alleviates the possibility of confusion between pilin-based filaments and those comprised of cytochromes. For example, heterologous expression of the gene for the PilA pilin monomer of *G. sulfurreducens* in *P. aeruginosa*, in place of the *P. aeruginosa* wild-type pilin, yielded pili with a diameter of 3.2 ± 0.2 nm (Liu et al., 2019), the diameter expected for filaments comprised of *G. sulfurreducens* PilA pilin (Reguera et al., 2005; Malvankar et al., 2014, 2015; Adhikari et al., 2016; Tan et al., 2017). The diameter of the wild-type *P. aeruginosa* pili were 4.8 ± 0.6 nm (Liu et al., 2019).

The conductance of the pili in the strain of *P. aeruginosa* heterologously expressing the *G. sulfurreducens* PilA pilin monomer was 20-fold higher than the wild-type *P. aeruginosa* pili (Liu et al., 2019). This result is consistent with previous studies that have demonstrated that *P. aeruginosa* pili are poorly conductive (Reguera et al., 2005; Liu et al., 2014; Lampa-Pastirk et al., 2016). Expression in *P. aeruginosa* of a synthetic pilin monomer with the same number of amino acids as the *G. sulfurreducens* PilA monomer, but with an increased abundance of aromatic amino acids, yielded pili with higher conductance than the filaments derived from the *G. sulfurreducens* PilA pilin monomer (Liu et al., 2019). This result is consistent with the previously demonstrated importance of aromatic amino acids in the conductivity of *G. sulfurreducens* e-pili (Vargas et al., 2013; Adhikari et al., 2016; Lampa-Pastirk et al., 2016; Steidl et al., 2016; Tan et al., 2017). Thus, the results of Liu et al. (Liu et al., 2019) demonstrate that the *G. sulfurreducens* PilA pilin monomer assembles into e-pili similar to those observed in wild-type *G. sulfurreducens* under conditions in which expression of OmcS filaments is impossible.

**Figure 3:**
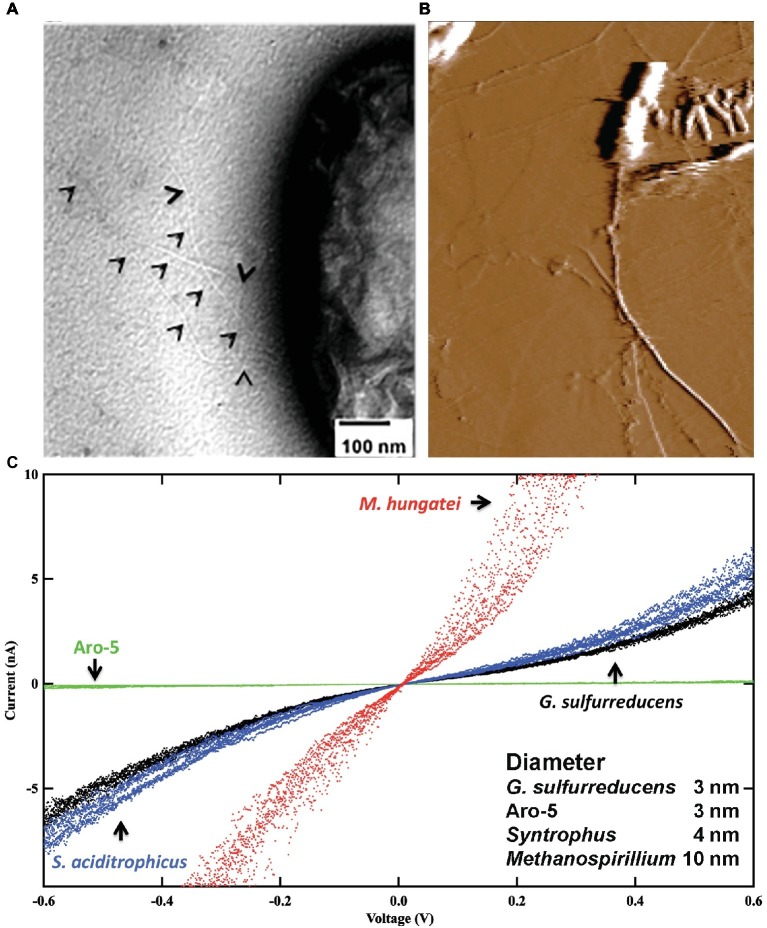
Conductive filaments in microorganisms that do not express outer-surface cytochromes. **(A)** Transmission electron micrograph of *Syntrophus aciditrophicus* expressing e-pili. Black triangles point to pili. **(B)** Atomic force micrograph of *Methanospirillum hungatei* expressing its e-archaellum. **(C)** Current-voltage response through individual e-pili or e-archaellum from current passing between a conductive AFM tip and the HOPG substrate on which the filaments were deposited. In all instances aliquots of cells were directly drop casted onto the HOPG from cultures with no chemical processing. *G. sulfurreducens* strains included a strain expressing the wild-type e-pili and the Aro-5 strain, which expresses a pilin monomer gene modified to yield poorly conductive pili. Image in **(A)** is modified with permission from (Walker et al., 2018b). Image in **(B)** is modified with permission from (Walker et al., 2019). Data in **(C)** is a redrawing of combination of data with permission from (Walker et al., 2018b, 2019).

Cytochromes were not detected in heme-stained preparations of *Syntrophus aciditrophicus* cell protein, yet *S. aciditrophicus* produces e-pili (Figure 3) that have a conductance comparable to those of *G. sulfurreducens* e-pili (Walker et al., 2018b). Additional analysis identified the likely pilin monomer gene in *S. aciditrophicus,* which was found to encode a high abundance of the aromatic amino acids expected to contribute to e-pili conductivity (Walker et al., 2018b).

The archaellum of Archaea is assembled from archaellin subunits, which show homology to the type IV pilins of bacteria (Poweleit et al., 2016; Albers and Jarrell, 2018). The conductivity of the archaellum of *Methanospirillum hungatei* was investigated (Walker et al., 2019) because: (1) the structure of this archaellum is known (Poweleit et al., 2016); (2) the archaellum is readily identified on the cell; and (3) *M. hungatei* does not express cytochromes. Analysis of individual archaella emanating from cells revealed (Figure 3) that they had a higher conductance than *G. sulfurreducens* e-pili (Walker et al., 2019). Conductance was attributed, at least in part, to a path of closely packed phenylalanine along the core of the structure.

### *G. sulfurreducens* Expression of Electrically Conductive-Pili

**Figure 4:**
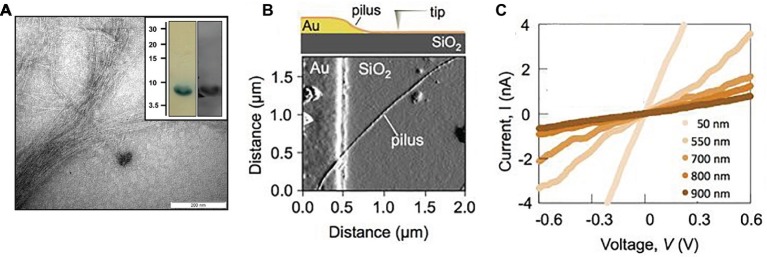
The individual e-pili in cytochrome-free preparations of *G. sulfurreducens* in which PilA pilin is the only protein are high conductive. **(A)** Transmission electron micrograph of purified e-pili; insert-after denaturation the PilA pilin monomer is the only protein detected on a SDS/PAGE Tricine gel (left) as verified with Western blot with anti-PilA antibodies (right). **(B)** Individual pilus on insulating silicon dioxide substrate and in contact with gold electrode as imaged with atomic force microscopy (AFM) as shown in schematic at top. **(C)** Current-voltage plots when a pilus was contacted at different distances from the gold electrode, demonstrating the ohmic-like conductivity of the pilus and the expected increased resistance with increased distance of electron transport. Image in **(A)** reproduced with permission from (Cologgi et al., 2011). Images **(B,C)** reproduced with permission from (Lampa-Pastirk et al., 2016).

There are multiple examples in which the electrically conductive filaments expressed in *G. sulfurreducens* appear to be comprised of the PilA pilin monomer, not OmcS. In an elegant study, the conductivity of individual filaments was documented in filament preparations (Figure 4) in which the PilA pilin monomer was the only protein detected (Lampa-Pastirk et al., 2016). The purification of the e-pili relied on a previously described method (Cologgi et al., 2011) in which detergent soluble materials, including OmcS, were separated from the insoluble PilA filaments with preparative gel electrophoresis (Cologgi et al., 2011). No iron was detected in the filament preparations with inductively coupled plasma-atomic emission spectroscopy and there were no absorption peaks at 406 and 528 nm, characteristic of *c*-type cytochromes. Denaturation of the pili yielded just one band on SDS page gels, the PilA monomer (Cologgi et al., 2011). The filaments reacted with a PilA antibody, detected with immunofluorescence (Cologgi et al., 2011). The conductivity along the length of individual PilA-based pili was 1–4 S/cm (Lampa-Pastirk et al., 2016). When a strain of *G. sulfurreducens* was constructed in which one of the tyrosines in PilA thought to be important in electron transport was replaced with an alanine the conductivity of the individual filaments decreased 5-fold. All of these results are all consistent *G. sulfurreducens* expressing a PilA pilin-based conductive filament.

In another study, some debris other than filaments were apparent in the preparation, but there was no report of cytochrome contamination (Ing et al., 2017). The PilA pilin monomer was only detected after the strong denaturation conditions previously identified (Cologgi et al., 2011) to be necessary to denature e-pili were employed (Ing et al., 2017). Results were presented showing PilA as the only protein in SDS PAGE gels. Whereas PilA was detected with liquid chromatography/mass spectrometry, no peptide residues of OmcS were detected (Ing et al., 2017). Films of the putative e-pili had an ohmic-like, linear current-voltage response (Ing et al., 2017). Bipotentiostat measurements recorded no redox peaks associated with cytochrome-mediated charge transfer (Ing et al., 2017). The conductivity of the filaments increased as the temperature was decreased, which contrasted with the decrease in conductivity expected with electron transport *via* electron-hopping between the hemes of cytochromes (Ing et al., 2017).

Filament preparations were less pure in the studies to be discussed next, in part because filament preparation methods were designed to have as little impact on the *in vivo* structure of the e-pili as possible. However, the presence of OmcS and other redox active molecules on the outer surface of *G. sulfurreducens* was well-known (Leang et al., 2003; Mehta et al., 2005, 2006; Reguera et al., 2005; Nevin et al., 2009; Inoue et al., 2010a,b; Qian et al., 2011). Therefore, studies were designed to account for the presence of OmcS and other potential contaminants. One important consideration in these studies was to ensure that the diameter of the individual filaments that were investigated was the 3 nm expected for e-pili.

**Figure 5:**
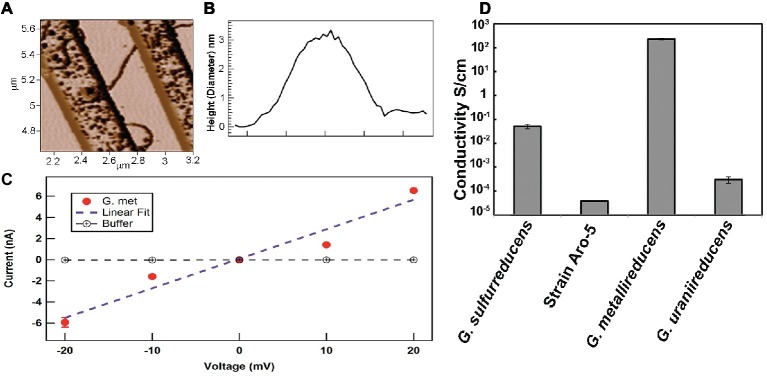
Conductivity of individual pili in strains of *G. sulfurreducens* expressing different pilin monomer genes. **(A)** Atomic force microscopy of pilus obtained when the pilin monomer gene of *G. metallireducens* was heterologously expressed in *G. sulfurreducens*. The pilus is laying on nano-electrodes for conductivity measurement. **(B)** 3 nm diameter (height) of the pilus like those shown in **(A)**. **(C)** Current-voltage response along the length of individual e-pili obtained when the *G. metallireducens* pilin monomer gene was heterologously expressed in *G. sulfurreducens*. **(D)** Conductivities along the length of individual pili obtained from expressing different PilA pilin monomer genes in *G. sulfurreducens*, as previously reported (Adhikari et al., 2016; Tan et al., 2016b, 2017). Images in **(A–C)** reproduced with permission from (Tan et al., 2017).

For example, the conductivity along the length of individual filaments from wild-type cells were compared with filaments from strain Aro-5 (Adhikari et al., 2016), a strain in which several aromatic amino acids in the PilA pilin monomer were replaced with alanine (Vargas et al., 2013). The Aro-5 strain displayed an abundance of extracellular OmcS comparable to wild-type cells (Vargas et al., 2013). The diameter of the filaments evaluated was 3 nm, consistent with PilA pilin-based filaments, but not OmcS filaments (Adhikari et al., 2016). At the physiologically relevant pH 7, the filaments from the wild-type were more than 1,000-fold more conductive than the filaments from Aro-5 (Figures 2, 5).

Also informative was the heterologous expression of the PilA pilin monomer from different *Geobacter* species (Figure 5). An example is the heterologous expression of the gene for the *G. metallireducens* PilA pilin in *G. sulfurreducens* (Tan et al., 2017). *G. metallireducens* is the closest known relative of *G. sulfurreducens* (Lovley et al., 2011). The *G. metallireducens* PilA pilin is highly homologous to the *G. sulfurreducens* PilA (Holmes et al., 2016). The *G. metallireducens* PilA is comprised of 59 amino acids compared to the 61 amino acids of *G. sulfurreducens*. However, the *G. metallireducens* PilA has a higher abundance of aromatic amino acids thought to be important in e-pili conductivity than the *G. sulfurreducens* PilA (Tan et al., 2017). When the *G. metallireducens* PilA gene is heterologously expressed in *G. sulfurreducens*, the electrically conductive filaments recovered are the same diameter (3 nm) as the filaments recovered from the control strain expressing the wild-type PilA (Tan et al., 2017); a diameter consistent with PilA pilin-based filaments, but not the 4 nm observed for OmcS filaments. Yet the filaments of the strain heterologously expressing the *G. metallireducens* PilA are 5,000-fold more conductive than the pili assembled from *G. sulfurreducens* pilin and also orders of magnitude more conductive than that reported (Wang et al., 2019) for OmcS filaments. In contrast, *G. uraniireducens* uses an electron shuttle for long-range electron transport and has a pilin with low aromatic amino acid abundance (Tan et al., 2016b). Heterologous expression of the *G. uraniireducens* pilin gene in *G. sulfurreducens* yielded pili with a 100-fold lower conductivity than *G. sulfurreducens* wild-type e-pili. The results from these studies and the expression of the synthetic Aro-5 pilin gene are readily explained if: (1) the filaments are comprised of PilA pilin monomer and (2) aromatic amino acid abundance of the pilin monomer plays an important role in the conductivity of pilin-based pili. These results are inconsistent with filaments comprised of OmcS.

Direct observation of filaments emanating from cells can avoid potential artifacts associated with purification procedures (Veazey et al., 2011). Scanning tunneling microscopy of filaments associated with cells revealed electron states indicative of a conductive material (Veazey et al., 2011). Accurate estimates of filament diameter are difficult with STM, but the filaments were estimated to be 3–5 nm in diameter (Veazey et al., 2011). This range is consistent with either e-pili or OmcS filaments. However, the filaments had an axial periodicity consistent with pilin-based filaments, not OmcS filaments (Veazey et al., 2011). Furthermore, electronic states routinely associated with cytochromes were not observed.

Another study on filaments emanating from cells, rather than outer-surface protein preparations, examined the filaments with electrostatic force microscopy (Malvankar et al., 2014). The filaments examined were 3 nm in diameter, as expected for e-pili, not the 4 nm diameter expected for OmcS. Furthermore, studies were conducted with the KN400 strain of *G. sulfurreducens*, which expresses very little OmcS (Yi et al., 2009). Electrostatic force microscopy demonstrated charge propagation along micrometer distances (Malvankar et al., 2014). In contrast, there was no charge propagation along pili from the Aro-5 strain of *G. sulfurreducens* (Malvankar et al., 2014). These results are consistent with charge propagation along e-pili, not OmcS filaments.

**Figure 6:**
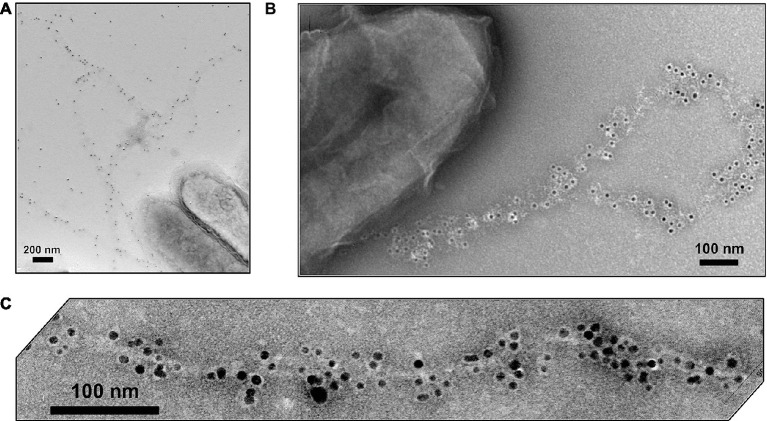
Transmission electron micrographs of immunogold **(A,B)** and Ni^2+^-NTA-gold **(C)** labeling of the e-pili emanating from *G. sulfurreducens* when the gene for a PilA pilin monomer in which the carboxyl end was amended with six histidines (His-tag) was expressed along with the wild-type pilin gene. The black, electron-dense particles are nano-gold particles (10 nm diameter, **A,B**; 5 nm, **C**) attached to the His-tag specific reagents. Images reproduced with permission from (Ueki et al., 2019).

*G. sulfurreducens* assembling its PilA pilin monomer into conductive filaments was obvious in a study in which short “peptide tags” were added to the carboxyl end of the monomer (Ueki et al., 2019). Tags evaluated were a “His-tag” (six histidines) and a “HA-tag” (YPYDVPDYA). The strain with the His-tag pilins expressed filaments (Figure 6) that reacted with immunogold reagents highly specific for the His-tag (Ueki et al., 2019). Addition of a gene for a pilin monomer with a HA-tag (YPYDVPDYA) on the carboxyl end resulted in a strain that expressed pili that contained both His-tag and HA-tag pilins, as determined with immunogold reagents. In both strains, the only filaments detected with transmission electron microscopy were immunogold labeled, suggesting a lack of OmcS filaments. Conducting probe atomic force microscopy revealed slight increases in conductance of the pili with peptide tags over that of the wild-type. The strains expressing the peptide-tagged pili produced current densities comparable to wild-type, confirming that the capacity for long-range electron transport had been maintained (Ueki et al., 2019). These results demonstrate that *G. sulfurreducens* produces electrically conductive pili from the PilA pilin monomer with no evidence of OmcS filaments emanating from the cells.

Examination of cells grown with Fe(III) oxide as the electron acceptor are especially relevant to the inquiry of expression of e-pili because only cells that contain genes for pilin that can assemble into conductive filaments are capable of reducing Fe(III) oxide without adaptively evolving strains to produce an electron shuttle (Vargas et al., 2013; Liu et al., 2014; Tan et al., 2016b). The filaments extending from cells grown on Fe(III) oxide have a diameter consistent with e-pili rather than OmcS filaments (Chen et al., 2019).

### Multiple Configurations for OmcS Localization on the Cell Outer Surface

**Figure 7:**
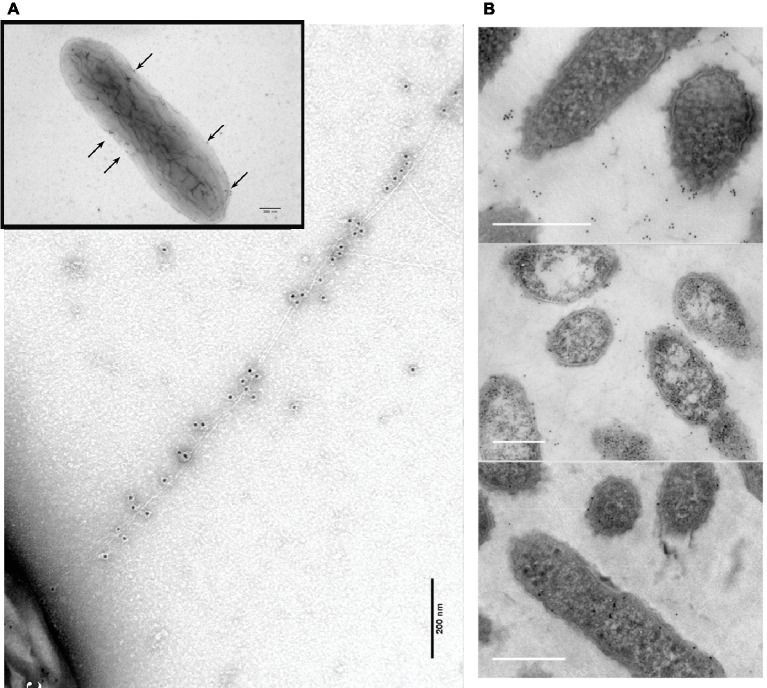
OmcS localization on filaments. **(A)** Transmission electron microscope (TEM) images of immunogold labeling demonstrating OmcS localization on cell surface in mid-log phase cells (inset) and along filaments in late-log phase cells. **(B)** TEM images of thin-sections of cells immunogold labeled for OmcS demonstrating: (top) OmcS at distance from the cells in wild-type cells, consistent with observations of labeling on filaments in whole cell preparations; (middle) OmcS associated with the outer cell surface in a mutant strain that did not produce PilA monomer; and (bottom) lack of OmcS labeling in an *omcS*-deficient mutant. Images in **(A)** reproduced with permission from (Leang et al., 2010). Images in **(B)** reproduced with permission from (Flanagan et al., 2017).

Immunogold labeling designed to investigate the localization of OmcS revealed that in early phases of growth with fumarate as the electron acceptor OmcS was associated with the cell surface (Leang et al., 2010). In late log and stationary phase, labeling for OmcS was also apparent along filaments extending from the cell (Figure 7). Notably, even in the later stages of growth on fumarate, only 26% of the fumarate-grown cells had filaments that were labeled with the OmcS immunogold technique (Leang et al., 2010). A higher percentage (56%) of Fe(III) oxide-grown cells were labeled. On filaments that were labeled there were often large gaps between clusters of gold labeling for OmcS. In wild-type cells (Leang et al., 2010), filaments that had sections that interacted with the OmcS antibody were often associated with another filament and individual filaments branched off from these associated filaments that were not labeled (Figure 7). Individual filaments emanating from cells that were also not labeled were observed in both wild-type cells (Figure 7) and a strain in which *omcS* was deleted (Leang et al., 2010).

At the time, these observations were made the possibility of OmcS filaments was under serious consideration (Tran, 2009), but the discontinuous immunogold labeling for OmcS along filaments was interpreted as association of OmcS with e-pili (Leang et al., 2010), an interpretation consistent with other results presented below. The alternative explanation is that inefficiencies in immunogold labeling resulted in the patchy distribution of OmcS antibody observed along the filament and that the labeled filaments observed were OmcS filaments. These uncertainties highlight the difficulties in interpreting the OmcS immunogold labeling and the need for alternative, more rigorous, methods to define the composition and structure of filaments emanating from *G. sulfurreducens*.

**Figure 8:**
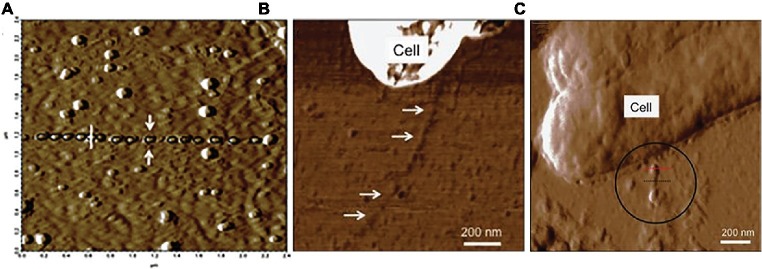
Atomic force analysis of possibility of OmcS association with e-pili from studies by Malvankar. **(A)** *c*-type cytochrome-like globules aligned along filaments. **(B)** lack of cytochrome-like globules on filaments of the *omcS* deletion strain. **(C)** *c*-type cytochrome-like globules on a strain of *G. sulfurreducens* in which the capacity for long-range electron transport was complemented in an *omcS* deletion strain with the heterologous expression of a multi-heme cytochrome gene from *G. bemidjiensis*. Image in **(A)** reproduced with permission from (Malvankar et al., 2012). Images in **(B,C)** reproduced with permission from (Yun et al., 2015).

Atomic force microscopy studies suggested that OmcS was associated with e-pili, rather than forming separate OmcS filaments (Figure 8). As in the immunogold studies, filaments associated with cells were investigated with a minimum of processing in an attempt to avoid any potential formation of artifact filaments (Malvankar et al., 2012). Atomic force microscopy revealed globules along filaments with an intermittent spacing that was similar to the OmcS immunogold labeling results (Malvankar et al., 2012). Long lengths of filaments lacked globules, but in some regions, globules were more closely associated (Malvankar et al., 2012). The filament-associated globules were absent in a strain of *G. sulfurreducens* in which the gene for OmcS was deleted (Yun et al., 2015). When a multi-heme cytochrome from another *Geobacter* species was heterologously expressed in *G. sulfurreducens* in place of *omcS,* the globules were again observed on the pili (Yun et al., 2015). This was significant because heterologous expression of that cytochrome gene also restored the capacity for Fe(III) oxide reduction that was lost when *omcS* was deleted, suggesting that the two cytochromes could promote Fe(III) oxide reduction in a similar manner (Yun et al., 2015). Studies on cells growing with Fe(III) oxide as the electron acceptor also noted filaments with the diameter of e-pili decorated with larger globules, consistent with the proposed localization of OmcS on e-pili (Chen et al., 2019).

Elemental mapping of filaments extending from *G. sulfurreducens* provided additional insights into the potential interactions of cytochromes and pili (Lebedev et al., 2019). Cells grown at 25°C with fumarate as the electron acceptor, a temperature that induces e-pili expression (Reguera et al., 2005), expressed filaments that atomic force microscopy indicated had a height (i.e. diameter) of 3.5 nm. The elemental composition of the filaments was consistent with that expected for filaments comprised of PilA pilin monomer (Lebedev et al., 2019). Energy dispersive X-ray spectroscopy (EDXS) detected iron, which appeared to be associated with protein “bundles” randomly distributed along the filaments (Lebedev et al., 2019). This pattern of putative iron-containing proteins along the filaments was similar to the patterns described above for immunogold labeling for OmcS (Leang et al., 2010) and the distribution of globules along apparent e-pili associated with OmcS expression (Figure 8). Cells grown at 30°C, a temperature that inhibits e-pili expression (Reguera et al., 2005), expressed filaments in which iron was more closely packed (Lebedev et al., 2019). However, the height of these filaments was 2–3 nm, too thin for OmcS. Further investigation to identify the composition these filaments is warranted.

As noted above, during the most active phase of growth on fumarate, OmcS was associated with the outer cell surface (Leang et al., 2010), demonstrating that OmcS is not always displayed as filaments extending at distance from the cell. In a similar manner, OmcS was associated with the cell surface in a mutant in which a gene near the PilA gene was deleted and PilA monomer was no longer expressed (Flanagan et al., 2017). The accumulation of OmcS on the outer surface, but a lack of filaments containing OmcS extending from the cell in this PilA-deficient strain (Figure 7) is consistent with the concept that OmcS is often associated with e-pili.

A potential explanation for the apparent association of OmcS with e-pili is the previously proposed (Lovley, 2006) role of OmcS as a conduit between cytochromes, like OmcB, which bring electrons to the outer surface, and e-pili. OmcS is known to be associated with the outer surface of cells (Leang et al., 2010; Flanagan et al., 2017) and could form chains along the outer surface between OmcB and e-pili. OmcS would need to establish a close association with e-pili for electron transfer. As e-pili are extended some OmcS may remain attached to the pilus and be transported at distance from the cell along with that region of the pilus.

**Figure 9:**
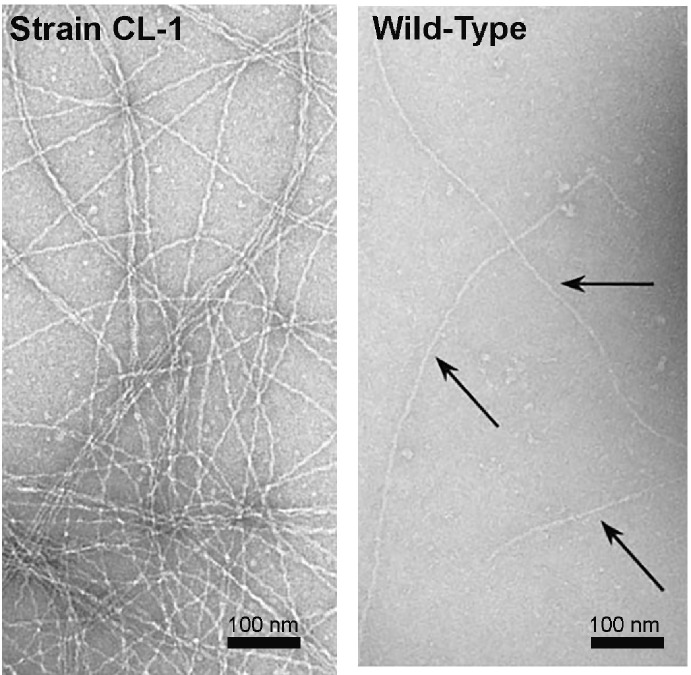
Transmission images of filaments from strain CL-1, which overexpresses OmcS, PilA, and exopolysaccharide (left) and wild-type cells (right). Images are a section of images that demonstrated the filaments emanating from cells. Images reproduced with permission from (Leang et al., 2013).

Wang et al. noted that some of the filaments in a previously published transmission electron micrograph of strain CL-1 (Leang et al., 2013) appear to have the morphology of OmcS filaments (Wang et al., 2019). Strain CL-1 is a mutant in which the gene for a protein with a PilZ domain was deleted (Leang et al., 2013). Proteins with PilZ domains have pleiotropic effects because of their post-translational mode of regulation of other proteins. Phenotypes in strain CL-1 included massive overexpression of OmcS and PilA, and the production of abundant exopolysaccharide not present in the wild-type cells (Leang et al., 2013). Side by side comparison of the transmission electron microscope images of the filaments emanating from cells from strain CL-1 and wild-type cells (Leang et al., 2013) demonstrate that some of the filaments associated with the CL-1 strain have a greater diameter and different morphology than the filaments associated with wild-type *G. sulfurreducens* (Figure 9). These results suggest that OmcS filament expression could be induced in *G. sulfurreducens* through mutation, but that OmcS filaments were not abundant in wild-type cells.

Fortunately, the distinctive differences in the structure of OmcS filaments and e-pili should make it possible to better evaluate the presence of these filaments emanating from cells in the near future with high resolution imaging. Much of the previous imaging of *G. sulfurreducens* has been on cells grown on fumarate, an electron acceptor with little environmental relevance. Imaging of cells grown under conditions in which long-range extracellular electron transport is required should aid in further evaluation of the expression and function of OmcS filaments *in vivo.*

### OmcS Filaments Are Not the Conduits for Long-Range Electron Transport Through Conductive Biofilms

Wang et al. (Wang et al., 2019) suggest that OmcS filaments are likely to play a role in long-range electron transport through electrically conductive biofilms. This conclusion ignores previous studies that demonstrated that OmcS was not required for *G. sulfurreducens* to produce high current densities or thick conductive biofilms (Malvankar et al., 2011, 2012).

Wang et al. (Wang et al., 2019) point out that in early studies (Holmes et al., 2006), an OmcS-deficient mutant of *G. sulfurreducens* produced less current than wild-type cells in “microbial fuel cells.” However, long-range electron transport is not necessary under these growth conditions because current densities are very low and the cells are in direct contact with the anode (Bond and Lovley, 2003; Reguera et al., 2006).

**Figure 10:**
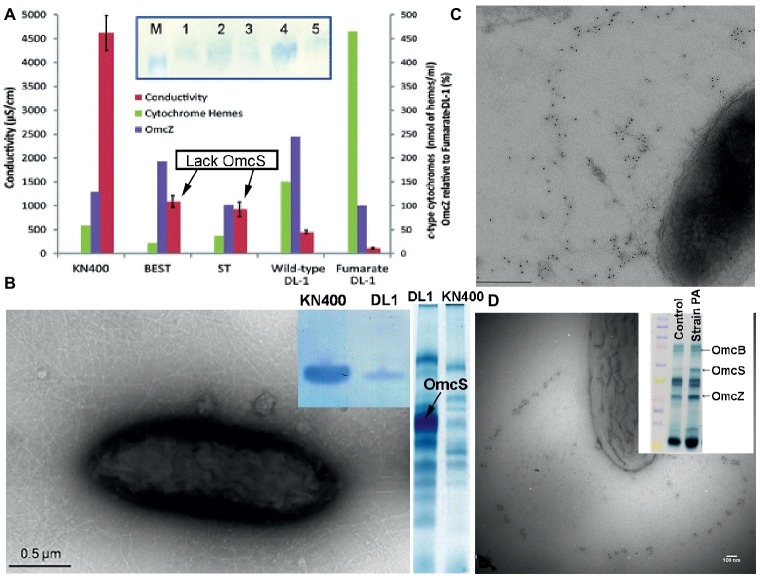
Multiple lines of evidence suggest that OmcS is not important in long-range electron transport. **(A)** Deleting OmcS *increases* biofilm conductivity. Shown is biofilm conductivity for wild-type cells (designated DL-1) grown with fumarate as an electron acceptor, as well as current-producing biofilms of wild-type cells (DL-1); a mutant strain in which genes for OmcS and OmcT were deleted (designated ST); and a strain in which genes for OmcB and OmcE were also deleted (designated BEST). Inset: Western immunoblot for OmcZ. M, marker; Lane-1, strain KN400; Lane-2, strain BEST; Lane-3, strain ST; Lane-4, wild-type; Lane-5, Fumarate-grown wild-type. **(B)** Transmission electron micrograph of strain KN400 demonstrating abundant filaments and SDS PAGE gels of (left) Western blot analysis for PilA pilin monomer of cells grown on anodes and (right) heme stain of outer surface cytochromes. **(C,D)** Immunogold labeling for OmcS in strain Aro-5 **(C)** and a strain of *G. sulfurreducens* expressing the pilin monomer of *P. aeruginosa* **(D)**. Both strains are deficient in current production and Fe(III) oxide reduction. **(D)** inset: heme-stained SDS PAGE gel of outer-surface proteins stained for heme demonstrating abundant OmcS in the mutant strain. Images are reproduced with permission from: **(A)** (Malvankar et al., 2012); **(B)** (Yi et al., 2009); **(C)** (Vargas et al., 2013); and **(D)** (Liu et al., 2014).

Long-range electron transport is required in bioelectrochemical systems in which current densities are ca. 15-fold higher and *G. sulfurreducens* produces thick (> 50 μm), electrically conductive biofilms, in which most of the cells donating electrons to the anode are not in direct contact with the anode. Deletion of *omcS* had no significant impact on current production in such systems (Nevin et al., 2009; Malvankar et al., 2011, 2012). In fact, deleting the gene for OmcS actually *increased* biofilm conductivity (Figure 10), even though the cytochrome content of the biofilm was decreased (Malvankar et al., 2012). These results demonstrate that OmcS filaments are not required for long-range electron transport and biofilm conductivity. The results contrast with the requirement for e-pili for higher current densities and conductive biofilms, as described in the next section.

### Multiple Lines of Evidence for the Role of Electrically Conductive-Pili in Long-Range Electron Transport Through Conductive Biofilms

There is substantial evidence consistent with e-pili supporting long-range electron transport in conductive *G. sulfurreducens* biofilms. For example, as noted above, *G. sulfurreducens* strain Aro-5 express pili that are much less conductive than wild-type e-pili because alanine was substituted for key aromatic amino acids in the synthetic Aro-5 pilin monomer (Vargas et al., 2013; Adhikari et al., 2016). Strain Aro-5 expresses abundant OmcS (Figure 10), including OmcS localized along filaments (Vargas et al., 2013). Yet biofilm conductivity of strain Aro-5 was ca. Ten-fold lower than wild-type (Vargas et al., 2013). This was accompanied by the production of low currents, consistent with only cells near the anode contributing electrons. Similar modifications to the aromatic content of the pilin monomer to reduce pili conductivity also greatly diminished current densities in another study (Steidl et al., 2016). These are the results expected if e-pili form conductive networks enabling long-range electron transport through current-producing biofilms.

The need for e-pili in order for cells to produce high current densities was also demonstrated with strains of *G. sulfurreducens* expressing the genes for pilin monomers from other microorganisms that assemble into poorly conductive pili. Heterologous expression of the pilin gene from *P. aeruginosa* in *G. sulfurreducens* yielded a strain with poorly conductive pili, but even more outer-surface OmcS (Figure 10) than the wild-type strain (Liu et al., 2014). Immunogold labeling revealed OmcS associated with filaments. However, as with strain Aro-5, the capacity for current production diminished compared with the wild-type strain. As discussed earlier, heterologous expression of the *G. uraniireducens* pilin gene in *G. sulfurreducens* also yielded poorly conductive pili (Tan et al., 2016b). Despite abundant OmcS on the outer surface, the *G. sulfurreducens* strain heterologously expressing the *G. uraniireducens* pilin monomer gene could not produce high current densities (Tan et al., 2016b).

Continuous propagation of *G. sulfurreducens* on anodes poised at a low potential yielded *G. sulfurreducens* strain KN400 (Yi et al., 2009). Strain KN400 expresses much less OmcS that the type strain of *G. sulfurreducens* and much more PilA (Yi et al., 2009). This was accompanied by the expression of abundant filaments (Figure 10), the capacity for significantly higher current production, and much more conductive biofilms, despite a much lower abundance of *c*-type cytochromes (Yi et al., 2009; Malvankar et al., 2011, 2012). These results, as well as the other studies described in this section, are all consistent with e-pili, not OmcS filaments, conferring conductivity to *G. sulfurreducens* current-producing biofilms.

### Electrically Conductive-Pili Expression Is Required for Fe(Iii) Oxide Reduction but There Are Substitutes for OmcS

As noted above, OmcS was initially discovered due to its important role in Fe(III) oxide reduction (Mehta et al., 2005). However, OmcS cannot accomplish Fe(III) oxide reduction without e-pili. The strains of *G. sulfurreducens* detailed in the previous section that heterologously expressed PilA pilin genes that yielded poorly conductive pili continued to express OmcS associated with outer-surface filaments, but were defective in Fe(III) oxide reduction (Vargas et al., 2013; Liu et al., 2014; Tan et al., 2016b). These results are inconsistent with concept of OmcS filaments serving as the primary conduit for long-range electron transport.

The capacity for Fe(III) oxide reduction in the OmcS-deficient mutant was restored with the addition of nano-particulate magnetite, (Liu et al., 2015). The magnetite was intermittently dispersed along the pili and attached to cells, in a manner similar to that observed for OmcS in immunogold labeling studies (Liu et al., 2015). Addition of magnetite to the *G. sulfurreducens* wild-type strain reduced transcript abundance of the gene for OmcS, suggesting that the cell regulated OmcS gene expression in response to the availability of an alternative that could serve the same function as OmcS. The magnetite was not simply substituting for hypothetical OmcS filaments because the presence of e-pili was required. Adding magnetite to cultures of *pilA*-deletion mutants did not enable Fe(III) oxide reduction (Liu et al., 2015).

### Observed Phenotypes Do Not Require OmcS Filaments Emanating at Distance From the Cell

As noted above, high resolution imaging of filaments emanating from cells will help further resolve whether OmcS filaments are a common feature of *G. sulfurreducens* growing under conditions that require long-range extracellular electron transport. *G. sulfurreducens* strains in which the gene for OmcS was deleted exhibit phenotypes that indicate that OmcS is important for some forms of extracellular electron exchange, but in none of these instances would it be necessary for OmcS to be arranged as filaments. For example, a strain in which the gene for OmcS was deleted was incapable of Fe(III) oxide reduction (Mehta et al., 2005). However, in these instances OmcS localized along the surface of the cell may facilitate electron transport to e-pili (Mehta et al., 2005; Lovley, 2006) or OmcS localized along e-pili may promote electron transport between e-pili and Fe(III) oxides (Lovley et al., 2011). Deletion of the OmcS gene inhibited electron uptake from zero valent iron, but the cells were in close association with the metal surface, and thus would not require long filaments of OmcS extending from the cell in order to establish electrical contact *via* OmcS (Tang et al., 2019). An OmcS-deficient mutant did not effectively grow as the electron-accepting partner in co-cultures with *G. metallireducens* as the electron-donating microorganism (Summers et al., 2010), but the requirement that *G. metallireducens* express e-pili for co-culture growth (Ueki et al., 2018) suggests that conduits other than OmcS are important for the long-range electron transport between the two species.

The physiology of *G. uraniireducens* may provide some further insight into the function of OmcS in extracellular electron transfer. OmcS homologs are only found in a few *Geobacter* species other than *G. sulfurreducens* (Butler et al., 2010). One of these is *G. uraniireducens*. *G. uraniireducens* is also one of the few *Geobacter* species without a gene homologous to the *G. sulfurreducens* PilA gene (Holmes et al., 2016) and its PilA assembles into poorly conductive pili (Tan et al., 2016b). Unlike *G. sulfurreducens* (Smith et al., 2014) and *G. metallireducens* (Nevin and Lovley, 2000), which possess e-pili and must directly contact Fe(III) oxide in order to reduce it, *G. uraniireducens* produces a soluble electron shuttle and reduces Fe(III) oxide without direct contact (Tan et al., 2016b). If as proposed (Wang et al., 2019) OmcS filaments, not e-pili, are an explanation for “the remarkable capacity of soil bacteria to transport electrons to remote electron acceptors for respiration” it would be expected that *G. uraniireducens* would express filaments of its OmcS homolog to facilitate long-range electron transport to Fe(III) oxides, but it instead produces an electron shuttle. In a similar manner, *G. uraniireducens* produces low current densities on anodes (Rotaru et al., 2015), further indicating e-pili rather than cytochrome filaments are essential for long-range electron transport. These results suggest that the OmcS homolog of *G. uraniireducens* is not assembled into long filaments enabling long-range electron transport.

### No Clear Role of PilA in OmcS Secretion

Wang et al. (Wang et al., 2019) state that “Multiple studies have shown that PilA is required for secretion of OmcS to the extracellular environment,” but there is substantial evidence that this concept is not correct. As noted above, a study of OmcS localization with immunogold labeling demonstrated that a mutant that did not express PilA still exported OmcS to the outer surface (Flanagan et al., 2017). In some instances deletion of the gene for PilA has resulted in lower recovery of OmcS in outer-membrane protein preparations (Liu et al., 2018), but this is not always the case (Richter et al., 2012; Steidl et al., 2016).

The suggestion by Wang et al. that “overexpression of PilA is also accompanied by overproduction of OmcS and filaments further suggesting that PilA is involved in secretion of OmcS filaments” is also not accurate. In multiple instances in which OmcS is expressed at higher levels the strains in fact express less PilA (Juarez et al., 2009; Summers et al., 2010; Shrestha et al., 2013) and there are also instances in which expression of more PilA is associated with lower OmcS expression (Yi et al., 2009; Malvankar et al., 2011).

### Culture Conditions Are a Key Consideration When Evaluating Protein Nanowire Expression

**Figure 11:**
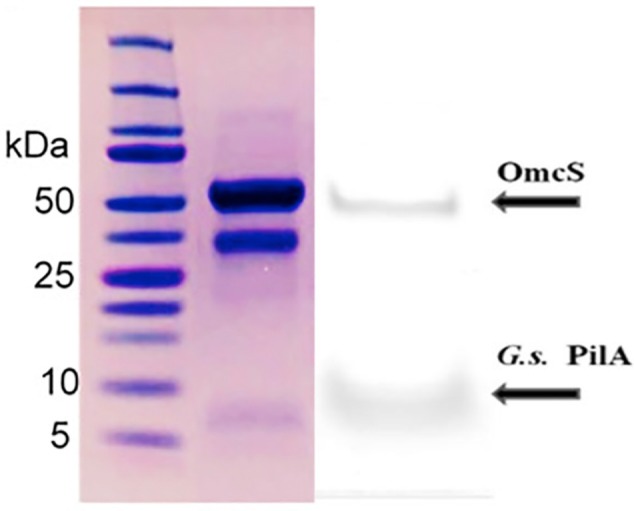
Comparison of the relative amounts of OmcS (at ca. 50 kDa) and PilA (at ca. 6 kDa) monomers in denatured filament preparations from *G. sulfurreducens* grown on anodes (left) in a “microbial fuel cell” or (right) a bioelectrochemical system that enables high current densities Image on left reproduced with permission from (Wang et al., 2019). Image on right reproduced with permission from (Tan et al., 2016b).

The claim by Wang et al. that OmcS filaments are the primary protein nanowires expressed by *G. sulfurreducens* rest largely on the abundance of OmcS filaments in their outer-surface protein preparations that were apparent with cryo-electron microscopy (Wang et al., 2019). However, PilA is barely detectable in their filament preparations (Figure 11). This contrasts with the abundance of PilA in filament preparations of wild-type cells prepared from cells grown as thick, electrically conductive biofilms (Tan et al., 2016b). In those filament preparations band densities for PilA in protein staining of SDS PAGE gels are at least equivalent to the density of OmcS bands (Figure 11). Given that the molecular weight of PilA is less than a fourth that of OmcS, this suggests that there are more PilA monomers than OmcS monomers in the outer-surface preparations. PilA is only detected when these filament preparations are treated with a harsh denaturation treatment demonstrating that the PilA monomers are present in e-pili (Tan et al., 2016b). In contrast, OmcS was recovered as monomers even with milder denaturation procedures (Tan et al., 2016b), as previously reported (Cologgi et al., 2011). These results are consistent with evidence from earlier studies that suggested that e-pili are abundant when cells are grown in thick conductive biofilms that require long-range electron transport.

One reason that Wang et al. recovered little PilA may be that the cells were grown in “microbial fuel cells,” a condition under which PilA gene expression is repressed and expression of the OmcS gene is upregulated (Holmes et al., 2006). As discussed in a previous section, long-range electron transport is not important to cells under these conditions because the cells are in close contact with the anode surface.

Growth conditions are also an important consideration in the interpretation of the conclusion by Wang et al. that OmcS filaments are more conductive than e-pili (Wang et al., 2019). This conclusion was based on the low conductivity of filaments recovered from a strain in which the gene for OmcS was deleted (Wang et al., 2019). Genetic manipulation of *G. sulfurreducens* and recovery of mutants are typically conducted with cells grown with fumarate as the electron acceptor (Coppi et al., 2001). Continuous propagation on fumarate selects against e-pili expression and can yield cells that produce abundant filaments that are not e-pili (Klimes et al., 2010). The filaments from the *omcS*-deletion mutant in the study by Wang et al. had diameters (1.7 nm) much thinner than those expected for e-pili, suggesting that some as-yet-unidentified filaments were being evaluated. In order to recover e-pili from the OmcS gene deletion mutant it is important to grow this strain under conditions in which e-pili expression is required, such as thick current-producing biofilms. When *G. sulfurreducens* is grown in this manner filaments with high conductivities with the diameter consistent with e-pili, not OmcS, are recovered (Adhikari et al., 2016; Tan et al., 2017).

## Conclusions

There is substantial evidence from multiple lines of investigation that the PilA pilin of *G. sulfurreducens* can assemble into e-pili and that e-pili facilitate long-range electron transport. An analysis of the current literature suggests that the available data does not support the conclusion (Wang et al., 2019) that the discovery of OmcS filament structure “explains the remarkable capacity of soil bacteria to transport electrons to remote electron acceptors for respiration and energy sharing”. As reviewed in detail above, even for the limited example of *G. sulfurreducens,* there are clear instances in which OmcS is not required for long-range electron extracellular transport, such as through thick, electrically conductive biofilms. *G. sulfurreducens* can also reduce Fe(III) oxides without OmcS filaments, but not without e-pili. The physiology of *G. uraniireducens* suggests that broad expansion of the cytochrome filament concept to “soil bacteria” is not borne out even within the *Geobacter* genus. There are also many bacteria capable of extracellular electron exchange that lack outer-surface cytochromes, but express e-pili (Sure et al., 2016; Walker et al., 2018a,b).

The assertion (Wang et al., 2019) that prior to the discovery of OmcS filaments, investigators routinely mistook OmcS filaments for e-pili ignores the great care that was taken in many studies to specifically rule out the possibility of cytochrome-based filaments. More work is required to determine if *G. sulfurreducens* expresses OmcS filaments extending at distance from the cell under conditions that require long-range extracellular electron transport and to evaluate how OmcS interacts with other electron transport components. High-resolution imaging of filaments associated with cells subjected to minimal processing is likely to be a productive approach to better understanding *in vivo* function.

The potential routes for extracellular electron transfer in *G. sulfurreducens* appear to be complex and the study of electron transfer is complicated by the potential pleiotropic impact of single gene mutations and the ability of the cells to rapidly adapt to genetic disruption of the removal of one potential route for extracellular electron transfer with the increased expression of the proteins for another (Lovley et al., 2011). Just one of many examples is the adaption of *G. sulfurreducens* to “go wireless” after long-term adaption to the genetic removal of the PilA gene by producing an electron shuttle (Smith et al., 2014). The study of how different electron transport components interact may best be studied in a “minimal *Geobacter*” in which the genes for all potential routes for extracellular electron transfer are genetically removed and the interaction of a small number of components reintroduced into the cell can be examined to determine the requirements for each extracellular electron transport route.

In-depth investigation of the mechanisms for extracellular electron transfer in *G. sulfurreducens* should be tempered by the consideration that an understanding of the fine details of extracellular electron exchange in one *Geobacter* strain may have little impact on many aspects of *Geobacter* ecology and applications in bioremediation and bioenergy (Lovley et al., 2011). Many of the extracellular electron transport components identified in *G. sulfurreducens*, including OmcS, are not conserved even in the closely related *G. metallireducens* (Butler et al., 2010; Smith et al., 2013). Different strains of *G. sulfurreducens* have substantial differences in their approach to extracellular electron transfer (Yi et al., 2009; Butler et al., 2012). Furthermore, there is a broad diversity of microorganisms capable of extracellular electron exchange; protein nanowires are just one strategy for electrical contact outside the cell (Shi et al., 2016; Lovley, 2017c,d). Understanding the broad concepts of e-pili function has helped identify diverse other microorganisms, including archaea, that may benefit from expressing conductive protein nanowires (Holmes et al., 2016; Walker et al., 2018a,b, 2019). The further development of an understanding that *c*-type cytochromes might form conductive filaments *in vivo* may be helpful in a similar way for identifying an enhanced diversity of microorganisms that electrically communicate with their extracellular environment.

Beyond their microbiological role, microbial protein nanowires are of interest because of the potential for biologically produced wires, or their biomimetic derivatives, to serve as conductive components in electronic devices (Lovley, 2017a; Creasey et al., 2018; Gutermann and Gazit, 2018; Ing et al., 2018). Studies with e-pili have demonstrated that it is possible to tune single wire nanowire conductivity (Adhikari et al., 2016; Tan et al., 2016a, 2017) and surface properties (Ueki et al., 2019) through the design of synthetic pilin monomers. The potential to heterologously express e-pili in other microorganisms (Liu et al., 2019) offers the possibility of mass production in a specifically designed chassis microorganism in which e-pili are the only filaments produced. OmcS filaments offer a nanowire structure and properties much different than e-pili, expanding the options for nanowire design. Exciting possibilities in the development of sustainable composite materials, wearable sensors, and memory and energy harvesting devices (Lovley, 2017a; Sun et al., 2018) can be expected to propel further study of microbially produced protein nanowires.

## Author Contributions

DL wrote the initial version of the manuscript after extensive discussions with DW who made substantive suggestions for subsequent revisions.

### Conflict of Interest Statement

The authors declare that the research was conducted in the absence of any commercial or financial relationships that could be construed as a potential conflict of interest.
